# Medical record: systematic centralization versus secure on demand aggregation

**DOI:** 10.1186/1472-6947-11-18

**Published:** 2011-03-22

**Authors:** Catherine Quantin, David-Olivier Jaquet-Chiffelle, Gouenou Coatrieux, Eric Benzenine, Bertrand Auverlot, François-André Allaert

**Affiliations:** 1 Inserm, U866, Dijon, F-21000, Univ de Bourgogne, Dijon, F-21000, France; 2 CHRU, Service de Biostatistique et d'Informatique Médicale, Dijon, France; 3 Bern University of Applied Sciences et Université de Lausanne, Suisse; 4 Institut TELECOM; TELECOM Bretagne; Unité INSERM 650 LaTIM; 5 Ceren Esc Dijon & Dpt biostat Ecole de Santé Publique Liège Belgique

## Abstract

**Background:**

As patients often see the data of their medical histories scattered among various medical records hosted in several health-care establishments, the purpose of our multidisciplinary study was to define a pragmatic and secure on-demand based system able to gather this information, with no risk of breaching confidentiality, and to relay it to a medical professional who asked for the information via a specific search engine.

**Methods:**

Scattered data are often heterogeneous, which makes the task of gathering information very hard. Two methods can be compared: trying to solve the problem by standardizing and centralizing all the information about every patient in a single Medical Record system or trying to use the data "as is" and find a way to obtain the most complete and the most accurate information. Given the failure of the first approach, due to the lack of standardization or privacy and security problems, for example, we propose an alternative that relies on the current state of affairs: an on-demand system, using a specific search engine that is able to retrieve information from the different medical records of a single patient.

**Results:**

We describe the function of Medical Record Search Engines (MRSE), which are able to retrieve all the available information regarding a patient who has been hospitalized in different hospitals and to provide this information to health professionals upon request. MRSEs use pseudonymized patient identities and thus never have access to the patient's identity. However, though the system would be easy to implement as it by-passes many of the difficulties associated with a centralized architecture, the health professional would have to validate the information, i.e. read all of the information and create his own synthesis and possibly reject extra data, which could be a drawback. We thus propose various feasible improvements, based on the implementation of several tools in our on-demand based system.

**Conclusions:**

A system that gathers all of the currently available information regarding a patient on the request of health-care professionals could be of great interest. This low-cost pragmatic alternative to centralized medical records could be developed quickly and easily. It could also be designed to include extra features and should thus be considered by health authorities.

## Background

Setting up an effective secure way to share information embedded in medical records between the different Health Structures (HS) involved in patients' care would greatly improve the quality of health care. This assertion is one of the main reasons for the development of Electronic Medical Records (EMR) over the last three decades. However, though the desire to provide professionals with access to all of the information related to patients is almost universally shared, in order to implement an effective system various difficulties have to be foreseen and overcome. This can be done early during the elaboration, since two architectural options can be compared: a single centralized, systematized, secure EMR system used by all Health Structures and including every patient, *vs*. a pragmatic secure system, able to retrieve information from the current non-centralized, non-standardized, non-structured EMRs and, most of all, only when needed and only for a particular request, *i.e*. an "on-demand" system.

The choice of approach depends on various criteria such as health care policies, governments' willingness, social and political context, *etc*. First of all, we are not aware of a country that has successfully implemented a standardized, centralized, secured, privacy-compliant and reliable EMR system. This is one of the many reasons why we have chosen to promote a non-centralized, non-standardized, on-demand system that relies on one main concept: to search for and retrieve distributed heterogeneous medical data. This approach is very close to what was proposed by Maro [1], and what is already effective in Israel (Clalit HMO and government hospitals), Pittsburgh (Pennsylvania - UPMC) [2] and is being implemented in Brussels (IRIS hospitals) [3] and Franche Comte, France (EMOSYST) [4].

We present below other reasons for this choice, and we aim to go further and propose in the methods section a practical and technical description of a system that overcomes many of the usual problems, especially the lack of standardization.

### Bottlenecks encountered in centralized systems

#### Centralization needs standardization, and standardization has needs

First of all, when no standard is available, it can be very difficult to create one. In many countries, harmonization of patients' identities is very difficult to achieve, and old previously stored medical data have to be re-indexed. The French concept of a Unique Patient Identifier (UPI) will effectively resolve this problem, but it is still in the initial deployment phase [5,6]. Furthermore, this UPI only concerns French nationals and other countries' initiatives, where they exist, would not use the same standards. In the face of such difficulties, the current strategy at the European level is to let each country define its own identification policy and to encourage interoperability between national information systems. Thus, a pragmatic solution that relies on data such as first name, last name and date of birth, which are present in all EMRs and does not require a UPI seems to be more appropriate and easier to achieve.

Moreover, besides patient identity, standardization also concerns many other fields and data harmonization of all health records at the national level can be difficult to achieve [7].

But the lack of standardization is not the only problem; even when the tools exist, they are not necessarily used. Regarding the standardization and structuring of the EMR system, a lot of time has been wasted trying to define a unique format for all doctors and all pathologies. The only domain where real harmonization has been obtained is "coding" - which is used to assess hospital activities, using the Systematized Nomenclature of Medical and clinical terms (SNOMED), and the International Classification of Diseases (ICD). However, though these terminologies are widely accepted and are now included in EMRs to record the activities of health-care facilities, they are not actually used for the daily management of patients' records in all European countries. The same applies to patients' drug treatments, which are key information, and for which there is the International Common Denomination (ICoD) used widely by the pharmaceutical industry, but which is still not used in a large number of prescriptions in countries where branded drugs are the most "popular".

#### The risks of centralization

The risks related to centralized records can be summarized as vulnerability and access management difficulties. For many years, the authorities have understood the risk of losing all of the data of a centralized system if the system is destroyed. Among other things, this conclusion led the US Department of Defense to create in 1969 the ARPANET, a network system that would be able to remain functional in case of a catastrophe. Regarding health data, the same approach can be applied and it would be obviously much safer to store such data in different places to ensure the protection of information, as it could be, for example, a target for terrorists who wish to destabilize a country by destroying or by pirating its health system and by divulging health information on citizens. Furthermore, hackers may see a centralized system as a challenge and try to gain access to a centralized patient EMR system and modify patients' medical information.

One could argue that centralized systems may appear easier to protect [8], by involving a team of security specialists devoted to implementing and enforcing security strategies for the entire structure, unified under a common set of principles. As an objection, it could be said that read/write access rights can be set up more easily [9,10] at the local level, *e.g*. by disabling write permission for all incoming connections from outside the local network.

Finally, to maintain the completeness of the EMR, every single actor has to be connected to the centralized system to notify it of every single operation he makes. Regarding the case of drug prescriptions, for example, the regulation of traffic load and security could be very difficult to manage.

In addition, to be effective, this kind of infrastructure requires a systematic process for every patient, without discrimination.

### On-Demand based Aggregation System: a true alternative

As centralized systems seem to be hard to build, to maintain and to protect, they cannot be an effective secure way to share information in the near future. In contrast, decentralized systems seem to be more flexible [1].

In our opinion, it seems feasible to set up a system that allows each doctor, with the authorization of the patient, to collect information on that patient from the different HS. Once the doctor has obtained the medical information via his medical information exchange application, he will have to synthesize the patient's medical history for his specific use, save it, and update it regularly.

The basic organizational advantage is that it could be operational rapidly, provided that problems of harmonization are reduced. The principle of decentralized management requires that the saved EMRs in the various HS remain in their unmodified state in terms of content and structure. Even though the data are not standardized, several items or fields such as first name, last name and date of birth exist in each patient's EMR, regardless of the rest of information. Identifying and picking a specific EMR using such data can thus be considered safe and does not require any additional indexing.

The second benefit is that this job has to be done only when needed, which means, first, that the workload will be distributed among health professionals and then that the task will only be necessary if a health-care professional requires information.

With this in mind, we propose a system that stems from our previous work on Medical Search Engines (MRSEs), which are able to aggregate Patients' Health Information on-demand.

A practical solution describing the flow of information and how the system can achieve its task in a secure, privacy-compliant way is explained below.

## Methods

### General description of the Medical Record Search Engine system

When a patient and his/her doctor want to gain access to the patient's medical data, scattered among the servers of various hospitals or clinics, they first have to connect to an electronic server and identify themselves. The identification of the doctor should be based on strong authentication credentials. Typically, the doctor might use a token activated with a PIN code (or with biometrics) to give the answer in a challenge-response protocol. The identification of the patient could be based on a smartcard (E-health card) for example. Once the authorization is granted, Medical Record Search Engines (MRSE) will securely gather medical information about the patient in a privacy-compliant way and transfer it to the Medical Practitioner (MP).

Two main points are considered. First, all of the retrieved information will be gathered by the hospital's system before being transferred to the MP's office, *i.e*. the management system requests information without directly reading the provider's local information. More clearly, all EMRs are kept and managed in a decentralized way in the "local" HS, recorded according to the system provided by the health structure's software and identified with the usual identifiers (first name, last name and birth date) which are present in all EMRs. Secondly, the patient's privacy is protected by using a pseudonymous code (derived from the patient's identity). All communications are encrypted.

### Entire routine procedure

**Figure 1:**
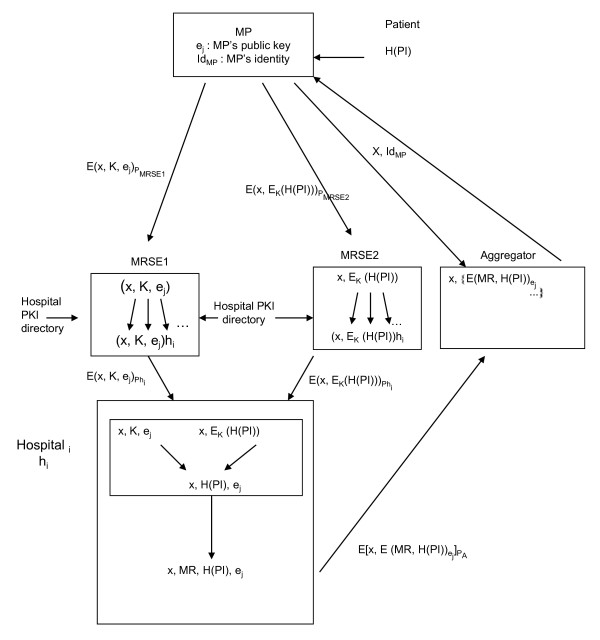
**Medical Record Search Engines' Procedure**.

To gain access to a patient's medical records, the proposed procedure can be described in seven steps (*cf*. Figure 1).

#### First step: Pseudonymization of patient's identity

During a consultation between a MP and a patient, the MP enters all the components of that patient's identification. This information related to the patient's identity will be "anonymized", using a robust cryptographic hash function to provide a Hashed Patient Identity called H(PI). The aim of this algorithm is to obtain a pseudonymous code, but, hopefully, always the same one for a given individual in order to link all of the information concerning any given patient.

#### Second step: Sending the request to the two MRSEs

When a MP wants to request a patient's information scattered in other HS, he has to send a request to two MRSEs and authenticate both himself and the patient. Exchanges between the MP and the MRSEs are protected by using an asymmetric encryption algorithm (like the RSA encryption). In this communication, the public keys (P_MRE1 _and P_MRE2_) of the MRSEs are used by the MP.

The MP sends a request called "x" to the two Medical Record Search Engines MRSE1 and MRSE2. The system hinges on the prevention of communication between MRSE1 and MRSE2. As seen in Figure 1, the information sent by the MP is split between the MRSEs. The purpose of MRSE1 and MRSE2 is to guarantee the confidentiality and the privacy of the request during its transmission.

MRSE1 receives three elements:

a) x, the number of the request,

b) K, a session key,

c) e_j_, the MP public key.

MRSE2 receives two elements:

a) x, the number of the request,

b) E_K_(H(PI)), the hashed patient identity H(PI), previously symmetrically encrypted by the MP with the session key K.

Through this procedure, MRSE2 is unable to access the pseudonymous patient identifier as it does not know the session key K, which has been transmitted to MRSE1. This prevents MRSE2 from finding the identity of the patient through, for example, a dictionary attack. In order to preserve patient's privacy, MRSE1 and MRSE2 are not able to communicate with each other.

#### Third step: Request transmitted to all HS by the two MRSE's

To transmit the request to the hospitals, both MRSEs first have to decrypt the messages sent by the MP, using their own private keys.

Then MRSE1 and MRSE2 consult an HS' directory in order to forward the request to all HS to which they are connected. MRSE1 and MRSE2 sign their respective part and send it to the hospitals. The requested information is encrypted with the HS' public keys.

#### Fourth step: Search for the patient's EMR at the HS' level

Locally, each HS decrypts messages issued by MRSEs. Then, they also decrypt the pseudonymous patient identifier (H(PI)) with the session key K.

Each hospital 'h_i_' can then search for medical records corresponding to this pseudonym (comparing it with hashed identities of the patients hospitalized in h_i_). If this search is successful, i.e. if one corresponds to the previously received H (PI), the corresponding patient's EMR will be gathered before being sent to the aggregator.

#### Fifth step: Transfer of the results of the request to an aggregator

This step consists in sending to an aggregator a record containing three elements:

a) the number of the request, x

b) the hashed patient identity, H(PI)

c) the patient's EMR, digitally signed by hospital h_i _with an electronic signature.

This electronic signature allows non repudiation and verification of the integrity of the message. To ensure transmission security, confidential medical information such as the hashed patient identity H(PI) and the patient's EMR are asymmetrically encrypted with the MP public key e_j_. The MP is the only one who can decrypt this confidential medical information with his private key.

#### Sixth step: Gathering all patient information at the aggregator level

The aggregator collects information received from all HS and gathers all the results of the same request x. These results are sent to the MP, after a challenge-response authentication procedure. The MP will be then able to decrypt these results with his own private key.

## Discussion

Regarding security, MRSEs are platforms that never have direct access to the database of the local systems of the HS as it is the HS itself which makes the requests: the on-demand system could not be used to alter or destroy local information.

Regarding privacy, MRSEs are platforms where encrypted information is temporarily stored before being passed on. MRSEs do not store any EMRs but may keep logs of transactions. Furthermore, MRSE1 does not manage patient data, and MRSE2 only manages pseudoanonymous encrypted data. Hence, we also propose that MRSE1 and MRSE2 are not allowed to communicate with each other, and that they must be hosted in different locations under different responsibilities to ensure privacy.

Regarding feasibility, as the system relies on « as is » data, no modifications, especially no standardization, is needed. Although the absence of a need for a new unique identifier is a major advantage, it may raise some discussion about doubloons and collision risks.

Regarding doubloon risk (i.e. losing some information by not being able to link information concerning the same patient) it is, most of the time, due to a typo, and it is important to understand that the problem remains even in local EMR. However, in the on-demand MRSE system, it is possible to reduce such errors by implementing tools using phonetic algorithms as described in a previous papers [11] or other robust transformations like in [12] or in [13]. To improve linkage quality, it is also possible to envisage that the MP sends not only one pseudonymous identifier per patient but a list of pseudonymous partial identifiers for each patient. The creation of each pseudonymous partial identifier can be based on the different combinations of first names, last names and dates of birth. This could be very helpful in various situations, such as in patients with two last names (*e.g*. married women or a child of divorced parents), or a patient who has two first names (*e.g*. "David Roger" or "John Paul").

Regarding collision risk, (*i.e*. mixing two EMRs from two different patients), although it is also related to EMR information and not the on-demand system, it implies that the MP has to check all information, eventually with the help of the patient. However, a feasibility test-based tool, relying on observed data and probabilistic modeling, could be implemented in our system involving MRSEs. For each record, this tool could manage a linkage probability level (high, medium or low), and the centralized aggregator, when transmitting the results of the request, could give a hierarchical order with high, medium or low probability levels, so as to help the MP in the validation process.

Also regarding the ease of use, as previously mentioned, the extra work would be distributed between the different Health Professionals, according to needs. In other words, the system will gradually develop, little by little, and rather effortlessly compared with a centralized EMR systems. Furthermore, this recursive aspect of the on-demand system means that it is ready for use now.

Some MPs may complain that they will have to read all of the different information to detect possibly false or missing information. Several counter arguments can be put forward.

First, since embedded information in the EMRs is enough for most of the needs of health professionals, gathering scattered information on a patient is, for routine care purposes, rarely necessary. Secondly, an enquiry on a patient's medical history is not necessary in the vast majority of cases. Thirdly, it has to be done only once (unless the patient frequently seeks treatment in different places, which is rare) and just needs regular updates. Finally, a doctor can, with the patient's consent, pass on gathered information to other doctors when this patient moves.

Furthermore, only a few patients (but it should be estimated in a survey) have been hospitalized in many different hospitals, and therefore, the number of different EMRs that MPs will have to summarize, consecutive to one request, will be one or two rather than ten or twenty.

Nevertheless, it is important to take into account that this synthesis has to be done in collaboration with the patient, who could provide great help in the management of his own records. For example, regarding the doubloon risk i.e. the risk of losing information, it is easy to ask the patient if he has been hospitalized in another place than at the hospitals that answered the request and provided the information. Similarly, regarding the risk of collision, (the amalgamation of two or more EMRs from different patients), it is usually easy, except in emergency situations, to ask the patient if he has really been hospitalized at all the hospitals that answered. The situation is less easy if the records provided came from the same hospitals, but here again the patient should be able to say if the date of the stay and the disease recorded correspond to him or not. Furthermore, the hierarchical classification proposed, based on probability levels could help to clear up this kind of situation.

Regarding the efficiency, it could be useful to reduce the amount of data gathered, in order to make it easier to handle. Therefore, we planned to add a selection criteria tool to the basic request as in any kind of search engine. The criteria could relate to a time period, a list of hospitals, a clinical department, a clinical event, the pathology about which the MP requests precise information, *etc*. However, the information, even when reduced in quantity, will need to be analyzed and reorganized by the practitioner.

## Conclusion

In this paper, we have discussed the interest of a pragmatic solution to gather information about a patient's history that could be operational in the present context of information storage. Thus, we propose a data-secure, on-demand system that retrieves and aggregates information using Medical Record Search Engines, which could be a real alternative to centralized EMR management. Then, as it would be much less expensive and avoid the need for major reorganization of any kind of the medical archives, it offers a concrete solution that is ready for use and easy to set up in a short time. Furthermore, the implementation of various tools to improve the ease of use and the efficiency of the on-demand system has also been discussed.

A regional level could be the right level to set up an experiment to test this proposal.

## Competing interests

The authors declare that they have no competing interests.

## Authors' contributions statement

CQ and DOJC thought up and designed the method.

CQ, DOJC, GC, EB and FAA contributed to writing the paper and revised it critically for important intellectual content. All the authors gave final approval of the version to be published.

## Pre-publication history

The pre-publication history for this paper can be accessed here:

http://www.biomedcentral.com/1472-6947/11/18/prepub
